# The Story of the Insane from Year to Year

**Published:** 1892-06-04

**Authors:** 


### The Story of the Insane from Year to Year.

(Fourth Series.)

### ILLINOIS HOSPITAL FOR THE INSANE AT KANKAKEE.

This asylum, in point of size, will vie with all but our very largest English asylums. It was built in 1878, and it now contains 1,675 beds. The cost inclusive has been a trifle over 787 dollars per bed, while the building, exclusive of land and furniture, cost nearly 595 dollars per bed. As the dollar is worth about 4s. 2d of our money it will be seen that the asylum has cost about £123 per bed. It is built in blocks, these being entirely detached from each other, and there are about twenty-five separate buildings—some of the blocks containing as many as 107 patients, and some as few as 40. The Medical Superintendent is assisted by six medical officers, one of these being a lady physician. Dr. Davey is as strongly opposed as we are to any part of an asylum exceeding two stories, and he seems very favourably inclined towards the detached block, or cottage system as it has been called. Whatever advantages this system may possess it does not seem to increase the recovery rate, for we notice that of 1,340 admissions only 205 were discharged recovered, and although 109 were discharged "much improved," even the addition of these to the recoveries would be far below our English average. The medical staff is numerous, and the employment and recreation of the patients seem to be most carefully attended to.

### SOUTH AUSTRALIAN HOSPITALS FOR THE INSANE.

One of these is situated at Adelaide, and contains 235 patients; the other is at Parkside, and contains 582, making a total of 817, and including all the registered insane, whether private or pauper, in the colony of South Australia. Taking both asylums together, the year's admissions amounted to 239, the recoveries to 80, and the deaths to 63. As usual in colonial asylums, the admissions show a strange mixture of nationalities. Here we have Aboriginal Australians, Australians, English, German, Irish, Italians, Scotch, and Swedes. The percentage of recoveries on admissions was 33·4, which, taking into consideration the nature of the cases admitted, must be looked on as satisfactory, although lower than our English average. The death-rate stands at 7 9, which is also considerably lower than our average. On referring to Table 20, we find only three of the deaths were caused by phthisis. Dr. Cleland tells us that eight male attendants and two female attendants were suddenly attacked by patients; and he further tells us that he is "not in the habit of expressing sympathy" with the attendants in these cases, but rather of reprimanding them for not being on the alert. Here we do not wholly agree with Dr. Cleland. It is easy enough for a medical officer to talk in this strain; but if he were shut up with lunatics for twelve hours a day, he would find that he often needed sympathy, and that however desirable it was to be always on the alert it was difficult in practice. For one attack by an attendant on a patient there are a hundred by patients on attendants. The former are worked up into sensational newspaper paragraphs, while the latter are unheard of beyond the asylum. No workers need more sympathy than asylum attendants.

### FRIENDS' ASYLUM, PHILADELPHIA.

This institution was opened in the year 1811. It was built by the Society of Friends in Pennsylvania, and originated in a desire to provide more kind and sympathetic treatment of the insane than was at that time general. We extract the following particulars from the Medical Superintendent's seventy-fourth annual report: "Twenty-five men and thirty-two women were admitted during the year. Fourteen men and twenty-six women were discharged, of whom seventeen were recovered, and two men and one woman died. There were 114 patients in residence at the close of the year." Dr. Hall points out the advantages of early treatment, and says: "The benefit to be derived in removing a patient from his surroundings in the early stage of this disease, and the restraining influences exerted by coming in contact with strange faces, are points the importance of which cannot be too forcibly dwelt upon. Often does convalescence date from the change thus made." These views have been expressed times without number on this side of the Atlantic; but they have obtained little credence and have been followed by small results. The staff consists of Medical Superintendent and an Assistant Medical Officer; and a lady physician is attached to the staff, though not actually resident.

### THE NEW BRUNSWICK ASYLUM AT ST. JOHN.

This institution contained 442 patients at the close of the year. There were 112 admissions, 47 recoveries, and 42 deaths. The recoveries come out at 42·05 per cent. on the admissions, and the deaths at 10·63 per cent. on the average number resident. Table X. shows the causes of insanity in those admitted since the year 1875, and we notice that hereditary tendency was the cause in 330 instances, and drink in 165. During the same period there were 479 deaths, and phthisis accounts for 90 of them. The total admissions were 1,883, and among them we find one patient each from Cuba, Norway, Spain, Wales, and Austria, while Ireland sent 294, Scotland 33, England 46, Germany, 3, and the United States 23; a collection which Dr. Steeves probably looks on as more varied than pleasing.

